# Sexuality and Affection among Elderly German Men and Women in Long-Term Relationships: Results of a Prospective Population-Based Study

**DOI:** 10.1371/journal.pone.0111404

**Published:** 2014-11-04

**Authors:** Britta Müller, Christoph A. Nienaber, Olaf Reis, Peter Kropp, Wolfgang Meyer

**Affiliations:** 1 Institute of Medical Psychology and Medical Sociology, Medical Faculty, University of Rostock, Rostock, Germany; 2 Medical Center Rostock, Department of Cardiology and Angiology, Rostock University Hospital, University of Rostock, Rostock, Germany; 3 Clinic for Child and Adolescent Psychiatry, Rostock University Hospital, University of Rostock, Rostock, Germany; 4 Queen Mary University of London, Barts and the London School of Medicine and Dentistry, London, United Kingdom; Brock University, Canada

## Abstract

Satisfaction with sexual activity i.e. sexual satisfaction and the importance of sexuality and affection were analysed using data from the German “Interdisciplinary Longitudinal Study of Adult Development” (ILSE). At three measurement points, 1993–1995, 1997–1998, and 2004–2006 i.e. subjects' ages of 63, 67, and 74 years, participants' reports about their affection and sexual activity were collected. The sample of completed records used for this study consisted of 194 urban non-institutionalised participants, 68% male, all living with partners. Median levels of sexual satisfaction were reported, fluctuating between the measurement points of ages 63 to 74. Between baseline, first and second follow-up no differences were found in levels of sexual satisfaction, though at measurement points age 63 and 67 women were more satisfied than men. When measured at age 74, affection was given a higher priority than sexual activity. Although men and women reported similar priorities, sexual activity and affection were more important for men than for women. Satisfaction within the relationship can be predicted by the importance of affection, but not by that of sexual activity. Our results confirm the thesis of the ‘second language of sexuality’: for humans in their later years affection seems to be more important than for younger individuals.

## Introduction

Cultural attitudes and concerns about sexuality vary between countries and even more so between Western ones and those of the developing world. In the former, sexuality and affection have long been looked at from a biomedical point of view [1]. Findings about the negative influences of hormonal disturbances, severe illness or side-effects of medication seem to support the idea that the need for intimacy and sexual activity in older age is not so important. This is reflected in the “deficit model of ageing”.

Due to recent demographic changes, particularly with old age becoming a prolonged period of life spent in partnerships, the situation of the elderly and their perception of their health and partnerships became the subject of in-depth studies. Among elderly couples sexual activity and affection have an important impact on their physical and psychological well-being [2].

Recent studies of elderly couples in western societies focus almost exclusively on their sexuality and their perception of it. Affection has rarely been looked at. The findings with regards to sexuality concentrate on sexual intercourse, because of the assumption that most of the sexually active couples prefer this form of sexual activity [3]. Studies using representative samples from the US population showed that among people aged 65–74 partnered sexual activity is more frequent among married men and women compared to those living on their own [4]. Among persons with a spouse or in another intimate relationship, men are more likely to be sexually active than women. However, this gender disparity is considerably greater in persons living without a partner [3], [5].

Also the decline in sexual activity over the course of life is more pronounced in single persons than in people with partner. The earliest signs of decline in sexual activity in couples can be found between the 5^th^ (aged 41–50) and 6^th^ (aged 51–60) decade of their lives. In this phase a reduction both in percentage of sexually active couples and the frequency of intercourse in active couples could be demonstrated [6]. This decline is closely related to physical and hormonal changes that can result in functional impairments. Further, social standards and attitudes to sexual activity in the post- reproductive phase have to be considered [7], [8], [9]. Beckman et al. [10] demonstrated, analysing data of four birth cohorts of 70-year-old Swedish men and women, that later birth cohorts reported higher frequencies of sexual intercourse, fewer sexual dysfunctions and higher rates of satisfaction with sexual activity than those from earlier birth cohorts. These results apply to both unmarried and married persons [10]. The second decline occurs between the 8^th^ (aged 71–80) and 9^th^ (81–90) decade [6]. In developed countries in this phase of life chronic diseases with effects on sexual functioning are widespread, for example hypertension and diabetes [5], [11], [12], [13]. Findings suggest that the risk of sexual impairment is greater in men than in women [5], [14], [15]. Further, stressful psychosocial situations seem to be more frequent between the 8^th^ and 9^th^ decade of life, when periods of care delivered by one partner with subsequent lower levels of sexual involvement become more likely [16].

Other than knowledge about sexual activity, very little is known about the intra- individual changes of satisfaction with sexual activity in individuals over 60 years old living with a partner. There are only a few studies, mostly showing inconsistent results. In the population-based longitudinal ‘Olmsted Study of Urinary Symptoms and Health Status among Men’ people between the ages of 40 and 79 were studied. It was demonstrated that men with a regular partner at the beginning of the study period (baseline) experienced a bigger decline than men without a partner. This is most likely due to higher baseline levels for men with a regular partner [13]. However, there are no corresponding longitudinal population-based studies of females in this regard. Heiman et al. [17], utilising a cross-sectional design, studied sexual satisfaction in couples in the USA, Brazil, Germany, Japan and Spain, participants aged between 39 and 70 years. Their results suggest a positive association between satisfaction with sexual activity and length of relationship in men; in women this association is even more pronounced. The results of Heiman et al. [17] were based on people living in a partnership between 1 and 50 years duration. However, the authors did not state whether sexual satisfaction increased with longer durations of relationships i.e. 40 years plus. The findings do not indicate a decline in satisfaction with sexual activity of men and women; however no indication with regard to stability versus decline was made. Heiman et al. [17] showed gender differences regarding sexual satisfaction in relationships existing for 40 years and longer: satisfaction with sexual activity was greater in women than in men.

Recent studies regarding physical contact in older age focus mainly on sexual acts. Little is known about day-to-day intimacy of couples [18], though it has been used to predict the perception of sexual activity: Heiman et al. [17] reported that touching and caressing by partners, kissing and cuddling in men and woman could be used as a predictor for satisfaction with sexual activity. Waite et al. [3] studied the extent of nonsexual intimate contacts in relationships based on data of the National Social Life, Health, and Ageing Project-NSHAP. The results show that 95.6% of men and 95.8% of women living with a spouse and aged 57-64 hug or hold the partner once a month or more. Within the ageing process the corresponding percentages decreased little: in men aged to 75–85 to 90.4%; in women to 90.0%. The item ‘once a month or more’, however seems to be only a general criterion without proof that these interactions are part of day-to-day living.

In our study we considered intimacy as a special way of expressing affection short of actual intercourse i.e. nonsexual intimate interaction. These expressions of affection include a wide variety of activities such as greeting the partner with an embrace, a kiss, a pat on the back, a cuddle, hug or a caress [3]. Though sexual activity is often related to affection, the latter can also be found in day-to-day life without sexual activity.

The purpose of this paper was to describe the subjective experiences of sexual activity and affection among men and women in Germany, born 1930–1932 and living in long-term relationships. The sample was based on the data of the German ‘Interdisciplinary Longitudinal Study of Adult Development’ (ILSE), a multicenter cohort survey of a population-based urban sample.

Our assumption was that the process of ageing modifies social interactions and behaviours and the following become more important than sexual activity: physical closeness, being in an intimate relationship, belonging together and being cared for. To start with we analysed the development of satisfaction with sexual activity from ages 63 to 74. We then studied how affection and sexual activity were perceived by participants aged 74 years. Thirdly we tested the influence of person- and relationship-related items on the satisfaction with the partnership.

Four hypotheses were tested:

1. Satisfaction with sexual activity in long-term relationships does not decrease with age

2. Women living with a spouse are sexually more satisfied than men with a spouse

3. In long-term relationships affection is given a higher importance than sexual activity

4. The importance of both sexual activity and affection predicts satisfaction with the relationship

## Methods

### Ethics Statement

The study was examined, positively voted and approved by the Ethics Committee of the Faculty of Medicine, Heidelberg University, and the Ethics Committee of the Faculty of Medicine, Rostock University. Written informed consent was obtained from the participants.

### Sample

The data used for this study were part of the pool of the German “Interdisciplinary Longitudinal Study of Adult Development” (ILSE). The study was funded by the German Federal Ministry of Family, Senior Citizens, Women and Youth (BMFSFJ), the Baden-Württemberg Ministry of Science, Research and Art (MWA) and the University of Rostock. ILSE, an ongoing multicenter cohort study, commenced in 1993, aimed at identifying individual, social and economic determinants of a healthy, ageing population. Inter- and intra-individual differences and changes occurring from middle to higher adult age are studied, as well as the influences of environmental factors, behavioral aspects, life-events, health behaviors and mental and physical health on well-being.

ILSE utilises a biographical approach analysing the effects of the participants’ perception of biographical key situations on performance and adaptation in later life. The design is based on the assumption that there are gender, cohort as well as systemic social influences. Two cohorts, pre-war born participants i.e. born 1930–32 and post-war born participants i.e. born 1950–52 were studied. Both lived through their childhood and adolescence in very different times of German history and went through their developments confronted with important historical events. Further, samples were drawn from West Germany (region of Heidelberg) and East Germany (regions of Leipzig and Rostock) thus enabling to study the effects of different political systems on processes of ageing.

Participants were identified by using their postal addresses, randomly chosen from the official government registry after implementation of the stratification criteria sex and cohort membership. As a result 1106 participants were recruited for ILSE, both cohorts comprised of 553 persons. Men (52%) and East Germans (55%) were slightly over represented. So far the sample has been analysed at 3 measurement points at which the participants were tested by multidisciplinary teams of medical doctors, psychologists, sociologists and sports scientists.

**Figure 1:**
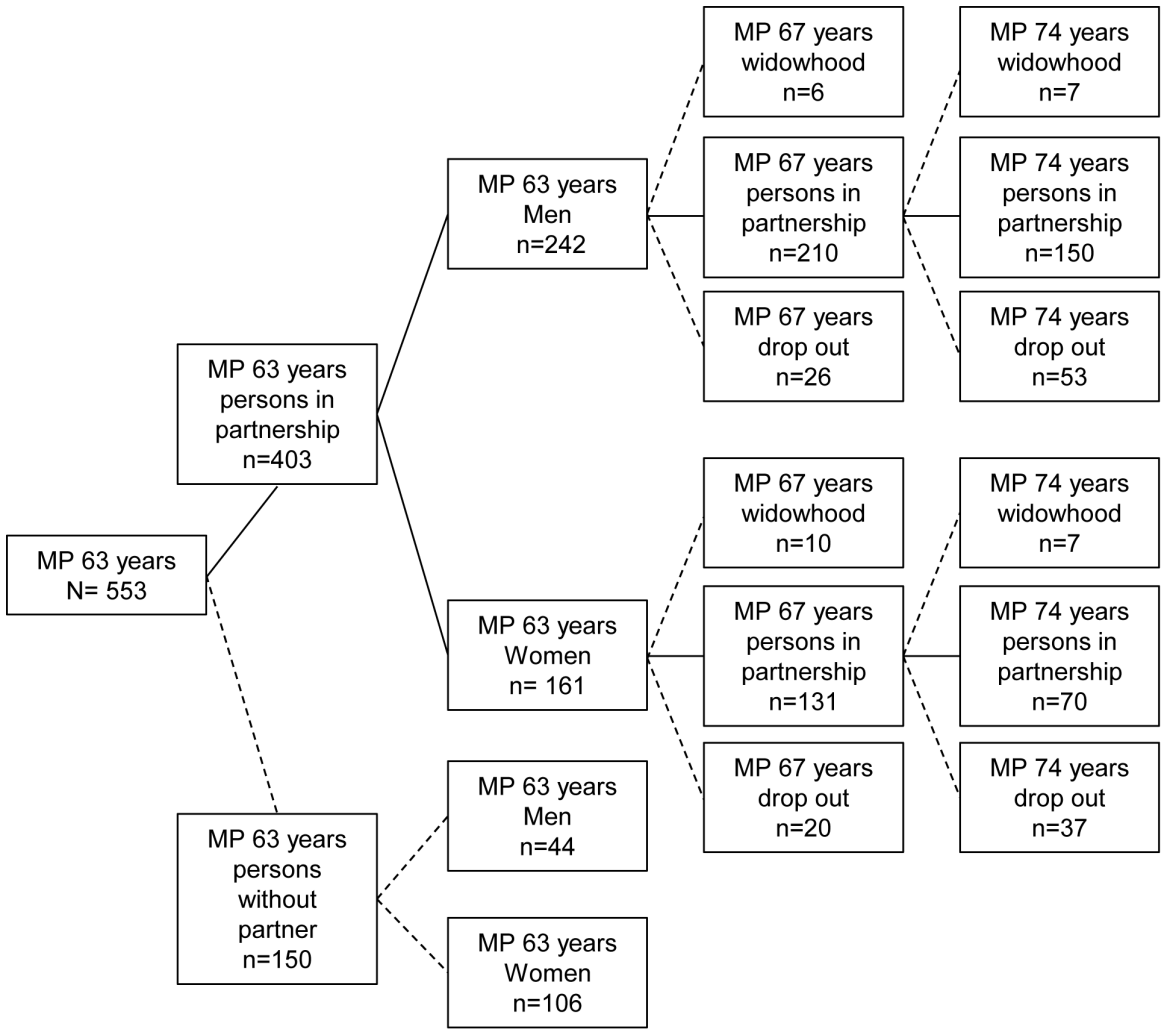
Selection process of the sub-samples at MP 63 years, MP 67 years, and 74 years. Persons who lived in relationship are shown with a solid line. Persons who dropped out or became a widow/widower are shown with a broken line.

Because the experience of sexuality in old age was a topic long neglected by research, it is worthy of a secondary data analysis. For this study data of the earlier birth cohort born 1930–1932 were used. This cohort was studied at three measurement points: first measurement point 1993–1995 i.e. baseline, average age 63; N = 553; second measurement point 1997–1998 i.e. first follow-up, average age 67, response-rate  = 89.9%, N = 497; third measurement point 2004–2006 i.e. second follow-up, average age 74, response-rate based on the baseline: 65.1%; N = 360. Only participants living in a relationship at all three measurement points and having provided a complete set of data about their sexual activity and affection at all three measurement points, were included in our study. At the first measurement point i.e. MP 63 years 74% of 553 participants were living in a relationship (Figure 1), 60% of those were male. At the second measurement point i.e. MP 67 years 341 persons still lived in a relationship, 62% of those were male. At the third measurement point i.e. MP 74 years 220 participants were in a relationship, 68% of those were male. 42% of the persons who had dropped out had died. A further 28% could not continue to participate because of ill health. The dropout analysis revealed that participants available at all three measurement points had better health at MP 63 (according to doctors’ assessments), better subjective health (according to their own assessments), better cognitive abilities and were less depressed than those who dropped out at the first or second measurement point.

Of 220 persons who lived in a relationship at MP 63, 67 and 74 years, we acquired complete data sets of 132 men and 62 women. This sample of 194 persons was studied. The proportion of men and women in our sample living in a relationship was representative of the German population [19], [20]. On average our female participants had male partners who were one year and nine months older. Looking at male participants, their female partners were on average three years and one month younger. So, being widowed is a less likely event for men than for women. All of the 194 participants had heterosexual partners and were married (Table 1). Most of the participants were living in long-term relationships. The averaged duration of the relationships was longer for women than for men, *U* = 3236, *p* = .019, *r* = .17. 87% of women and 86% of men were in their first marriage. Being married was not a criterion for participation. It can, however, be understood as an expression of social norms in the analysed cohort, for example the wish to legitimise a relationship by marriage. In addition, new relationships are less likely to be started by widowed women in advanced age [21]. In our sample of 194 participants we found differences between men and women regarding vocational training and occupation, which are typical for this generation. Men reported attending primary and secondary school and vocational training longer than women, *U* = 3216, *p* = .014, *r* = −.18. Their doctors ‘assessments of participants’ health were good overall and at none of the measurement points differed between men and women (see Table 1).

**Table 1:** Characteristics of Sample.

		Men	Women
		*n* = 132	*n* = 62
Average age (*M*, *SD*)	Baseline (1993–1995)	62.8 (0.9)	62.9 (0.9)
	First follow up (1997–1998)	66.8 (0.9)	66.7 (1.0)
	Second follow up (2004–2006)	74.1 (0.9)	74.0 (0.9)
Average duration of the current relationship MP 74 years (*M*, *SD*)		46.4 (8.0)	48.0 (8.0)
Duration of current relationship MP 74 years (*n*, %)	21 to under 30 years	10 (7.6)	4 (6.5)
	30 to under 40 years	8 (6.1)	5 (8.1)
	40 to under 50 years	56 (42.4)	15 (24.2)
	50 and more years	58 (43.9)	38 (61.3)
Number of marriages MP 74 years (*n*, %)	1	114 (86.4)	54 (87.1)
	2	15 (11.4)	8 (12.9)
	3	3 (2.3)	0
Average duration of education in years (*M*, *SD*)		14.1 (2.6)	13.1 (3.1)
Duration of education (*n*, %)	8 to 9 years	3 (2.3)	11 (17.7)
	10 to 15 years	90 (68.2)	36 (58.1)
	16 to 18 years	39 (29.5)	15 (24.2)
Occupation (*n*, %)	Higher-grade professionals	58 (43.9)	13 (21.0)
	Lower-grade professionals	16 (12.1)	15 (24.2)
	Routine non-manual employees	7 (5.3)	10 (16.1)
	Self-employed, artists	8 (6.1)	3 (4.8)
	Employed technicians; supervisors	10 (7.6)	2 (3.2)
	Skilled manual workers	29 (22.0)	5 (8.1)
	Semi-skilled/unskilled manual workers	4 (3.0)	14 (22.6)
Retirement (*n*, %)	Baseline (1994–1995)	100 (75.8)	59 (95.2)
	First follow up (1997–1998)	132 (100.0)	62 (100.0)
Physical health (*M*, *SD*)^a^	Baseline (1994–1995)	2.3 (0.8)	2.4 (0.9)
	First follow up (1997–1998)	2.5 (0.7)	2.6 (0.7)
	Second follow up (2004–2006)	2.5 (0.8)	2.5 (0.7)

a Assessment of physical health by a physician based on the history, physical assessment and blood tests using a six-point-scale: 1 =  very good, 2 =  good, 3 =  satisfactory, 4 =  sufficient, 5 =  poor, 6 =  very poor.

M: mean; SD: standard deviation.

### Measures

Data about ‘sexuality and affection’ were collected by a semi-structured interview. This was conducted to evaluate the participants’ current situation of life regarding health, housing, finance, job, partnership and social networks. Further, data were gathered about the participants’ subjective perception and individual summary of their lives so far and perspectives for the future. In addition, a detailed biography was obtained at the first point of measurement. That included details about how the participants perceived learning about sexuality in their adolescence, their first erotic encounters and sexual activity, and their sexuality in the first years of their relationship. In addition, from the second measurement point onwards they were asked about any changes in their life situation.

On average a semi-structured interview took one and a half hours. Interview techniques were honed extensively in training sessions of several days duration. Furthermore a concomitant quality control was implemented. All interviewers underwent a video-based certification process that required them to achieve at least 80% of the targets of an independently certified standard training [22].

The data regarding sexuality and affection described in our study were part of the interview part ‘relationship’. Data relating to satisfaction with sexual activity were collected at all three MP, however, the importance of sexual activity and the issue ‘affection’ was only explored at the MP 74 years. This was due to increased importance given to affection and sexual activity during the years the study ran. Our data about sexuality have to consider the different meaning of the word ‘sexuality’ in English and German: In the English language the term ‘sexuality’ is ambiguous although commonly used in research. Further, English speaking elderly people might be in a relationship they regard as sexual, i.e. in a marriage, but may not currently be sexually active. In the German language the word ‘Sexualität’, used in the study, refers more strongly to sexual activity than the English word ‘sexuality’. When asked what our participants understood as ‘Sexualität’ they indicated sexual activity in the sense of sexual intercourse. In the following we therefore refer to sexuality as ‘sexual activity’.

At MP 63 and MP 67 years the participants were encouraged to reflect on sexuality by the standardized question regarding sexuality was: ‘I now would like to speak about sexuality. Could you tell me how this is like in your partnership?’ Following that, the participants were asked to rate how content they were with sexuality in their relationship. Participants recorded their own answers by completing a 5 point Likert scale (‘How satisfied are you with sexuality in your partnership? 1 =  very poor; 2 =  poor; 3 =  satisfactory; 4 =  good; 5 =  excellent’).

At MP 73 years first the issue ‘affection’ was examined. Since we expected participants to know less about the concept of ‘affection’ compared to ‘sexuality’, verbal anchors for ‘affection’ were given in the standardized instruction: ‘Now I would like to speak about mutual proximity and affection in your partnership, for example embracing, holding hands, cuddling or kissing. Could you tell me something about this in your partnership?’ Answers contained very different behaviors, indicating that our participants understood the concept. For example everyday situations like guiding someone when walking were regarded as affection. Participants afterwards were asked to rate the importance of affection in their relationship and how satisfied they were with it. They recorded their answers in a 5 point Likert scale (‘How important is intimacy and affection in your relationship?’ 1 =  not at all important; 2 =  slightly important; 3 =  fairly important; 4 =  quite important; 5 =  very important; ‘How satisfied are you with intimacy and affection in your relationship?’ 1 =  very poor; 2 =  poor; 3 =  satisfactory; 4 =  good; 5 =  excellent’). After that we shifted to the issue ‘sexuality’ with the following question: ‘When you think about sexuality, could you tell me how this is like in your partnership?’ In the same way as for the previous issue ‘affection’ the questions to importance of sexuality and satisfaction with sexuality followed. Again, a list with answers was handed to participants. At the end of the interview part ‘affection and sexuality’ satisfaction with the relationship was rated by participants on a five-point Likert scale (1 = very poor; 2 = poor; 3 = satisfactory; 4 = good; 5 = excellent).

### Statistical analysis

In order to analyse the mean differences t-tests were used (Student's t-test; t-test for independent samples). Furthermore a repeated measurement analysis of variance and a multiple linear regression model were applied. The level of significance was set at p<0.05.

The data set for the study is available as a supplementary file for this publication (Table S1).

## Results

### Satisfaction with sexual activity

**Figure 2:**
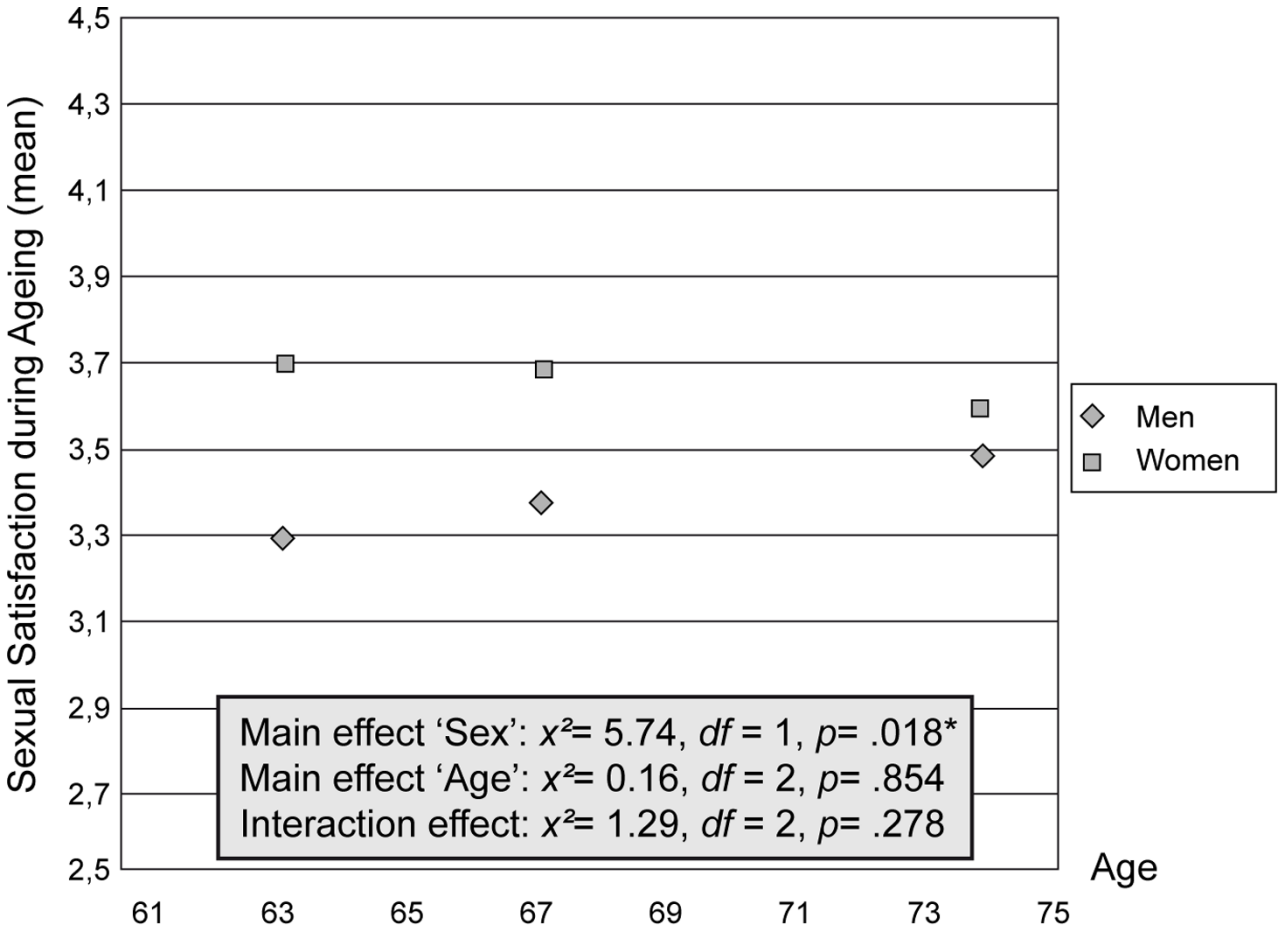
Satisfaction with sexual activity during ageing: mean-values, differentiated by gender. In the box are the results of the nonparametric repeated measures analysis of variance (Kruskal-Wallis-Test; Friedmann-Test). *p<.05.

During the observational period of 12 years satisfaction with sexual activity ranked at a median level between ‘satisfactory’ (3.0) and ‘good’ (4.0) (see Table 2). Differences between men and women were found *x^2^* (1)  = 5.74, *p* = .018 (see Figure 2) at baseline and first follow-up, none was seen at second follow-up. Women were more satisfied then men at MP 63 years (mean  = 3.69, *SD* = .88 vs. mean  = 3.29, *SD* = 1.14, p = .026) and at MP 67 years (mean  = 3.66, *SD* = 1.01 vs. mean  = 3.34, *SD* = 1.08, p = .054) (see Table 2). We did not find any differences of satisfaction with sexual activity between the three measurement points, neither in the total sub-sample nor in groups of either gender (Figure 2 and Table 3). Although levels of satisfaction between men and women became similar during the process of ageing, there was no significant age by sex interaction effect *x^2^*(2)  = 1.29, *p* = .278 (see Figure 2).

**Table 2:** Levels of satisfaction with sexual activity at MP 62, 66 and 74 years: total sample by gender.

	*M* (*SD*) [95% *CI*]			
Level of satisfaction with sexual activity^a^	Total *N* = 194	Men *n* = 132	Women *n* = 62	U-Test^b^ (Men-Women) *z-*value (*p*-value)
Baseline: MP 63 years	3.42 (1.08) [3.26;3.57]	3.29 (1.14) [3.09;3.48]	3.69 (0.88) [3.47;3.92]	−2.22 (.026*)
First follow up: MP 67 years	3.44 (1.06) [3.29;3.59]	3.34 (1.08) [3.16;3.53]	3.66 (1.01) [3.41;3.92]	−1.92 (.054)*
Second follow up: MP 74 years	3.52 (0.98) [3.38;3.66]	3.48 (1.01) [3.31;3.66]	3.60 (0.93) [3.36;3.83]	−.76 (.448)

a Level of satisfaction with sexual activity on a five-point-scale: 1 =  very poor, 2 =  poor, 3 =  satisfactory, 4 =  good, 5 =  excellent.

b Results of Mann-Whitney-U-Test, 2-Tail-Sig.

*p<.05.

M: mean; SD: standard deviation.

**Table 3:** Levels of satisfaction with sexual activity: results of the analysis of differences between the measurement points (MP).

Wilcoxon-Test^a^; *z*-value (*p*-value)			
Measurement points (MP)	Total *N* = 194	Men *n* = 132	Women *n* = 62
MP 63–74 years	−1.10 (.270)	−1.63 (.102)	−.61 (.539)
MP 63–67 years	−.43 (.664)	−.67 (.502)	−.28 (.782)
MP 67–74 years	−.96 (.337)	−1.44 (.150)	−.57 (.57)

a Results of Wilcoxon-Matched-Pairs-Signed-Rank-Test; 2-Tail-Sig.

### Sexual activity and affection

**Figure 3:**
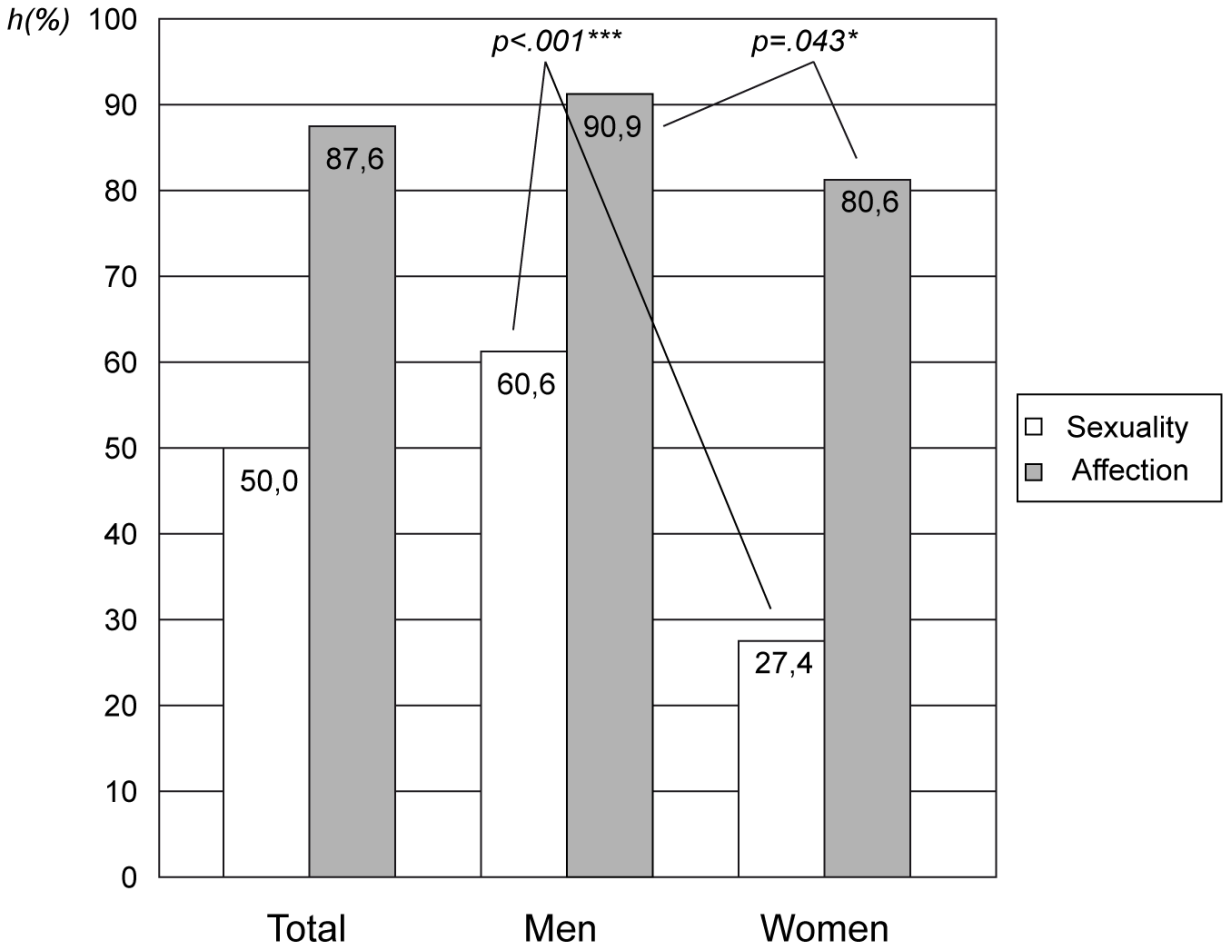
Percentage of elderly in the group MP 74 years to whom sexuality and affection is important. Summary of the relative frequencies ‘level 4’ (quite important) and ‘level 5‘ (very important). Results of Pearson's Chi-Squared-Test. ***p<.001.

With the beginning of the eighth decade of life sexual activity and affection ranked differently (see Table 4). Affection had a higher priority (mean  = 4.21; *SD* = .77) than sexual activity (mean  = 3.23; *SD* = 1.04). Although men and women had the same ranking, they differed regarding the level of importance: for men, sexual activity and affection were more important (sexual activity: mean  = 3.50, *SD* = .95; affection: mean  = 4.32, *SD* = .68) than for women (sexual activity: mean  = 2.66, *SD* = .99; affection: mean  = 3.98, *SD* = .90) (see Table 4). This difference became particularly evident when the answering options “very important” and “quite important” were collapsed into one; 60.6% of male participants reported that sexual activity would be important versus only 27.4% of women. However, for 90.9% of men and 80.6% of women affection played an important role in their life (see Figure 3).

**Table 4:** Importance of sexual activity and affection at MP 74 years (second follow up); total sample by gender.

	*M* (*SD*) [95% *CI*]			
Importance of…	Total *N* = 194	Men *n* = 132	Women *n* = 62	U-Test^c^ (Men-Women) *z-*value (*p*-value)
sexual activity^a^	3.23 (1.04) [3.08; 3.38]	3.50 (0.95) [3.34; 3.66]	2.66 (0.99) [2.41; 2.91]	*−5.12 (<.001* *** *)*
affection^b^	4.21 (0.77) [4.10; 4.32]	4.32 (0.68) [4.20; 4.44]	3.98 (0.90) [3.76; 4.20]	*−2.48 (.013* * *)*

a Importance of sexual activity on a five-point-scale: 1 =  not important at all, 2 =  hardly important, 3 = fairly important, 4 = quite important, 5 =  very important.

b Importance of affection on a five-point-scale: 1 =  not important at all, 2 =  hardly important, 3 =  fairly important, 4 =  quite important, 5 =  very important.

c Results of Mann-Whitney-U-Test, 2-Tail-Sig.

*p<.05;

***p<.001.

M: mean; SD: standard deviation.

### Satisfaction with the relationship

We found high levels of satisfaction with the relationship at age 74 years with a mean between ‘good’ (4.0) and ‘excellent’ (5.0) (mean  = 4.42; *SD* = .70). Men (mean  = 4.45, *SD* = .74) and women (mean  = 4.34, *SD* = .60) showed similar levels. The Mann-Whitney-Test did not indicate differences between men and women, *U* = 3489, *p* = .061, *r* = −.13. A multiple regression analysis was conducted using the following predictor variables: level of education, physical health, duration of relationship, importance of sexual activity and importance of affection. The dependent variable was satisfaction with the relationship. Regarding the total sample the model produced *R^2^* = .16, which was statistically significant, *F* (5,188)  = 8.32, *p*<.001. The explained variation within the group of women with *R^2^* = .30, *F* (5.56)  = 6.20, *p*<.001 was higher than that of men, *R^2^* = .11, *F* (5,126)  = 4.09, *p* = .002. Satisfaction with the relationship were predicted by the importance of affection in the total sample, *B* = 0.39, *t* = 5.69, *p*<.001, among men, *B* = 0.44, *t* = 4.28, *p*<.001 and among women, *B* = 0.33, *t* = 4.12, *p*<.001. Neither women nor men showed a relation between importance of sexual activity and satisfaction with the relationship. The results of the regression analysis are shown in Table 5.

**Table 5:** Summary of a multiple regression analysis to predict Satisfaction with relationship at MP 74 years.

	Total sample *N* = 194			Men *n* = 132			Women *n* = 62		
Predictors	*B*	*SE*	β	*B*	*SE*	β	*B*	*SE*	β
Years of education	−0.01	0.02	−.03	−0.01	0.02	−,01	−0.02	0.02	−.09
Physical health	−0.06	0.07	−.06	−0.05	0.08	−.05	−0.10	0.10	−.11
Duration of relationship	−0.01	0.01	−.10	−0.01	0.01	−.04	−0.02	0.01	.24*
Importance of sexual activity	−0.06	0.05	−.09	−0.11	0.07	−.14	0.03	0.07	.05
Importance of affection	0.39	0.07	.43***	0.44	0.10	.40***	0.33	0.08	.50***
*constant*	*3.61*			*3.31*			*4.30*		
***R^2^***	**.** ***16***			**.** ***11***			**.** ***30***		

*B* =  regression coefficient; *SE*  =  standard error; β =  standardized beta weight.

*p<.05;

***p<.001.

## Discussion

The ILSE study covered a surveillance period of 12 years. The participants, born between 1930 and 1932 and in good mental and physical health, were examined at the measurement points aged 63, 67 and 74 years. The satisfaction with sexual activity among men and women remained stable during the study period. We regarded hypothesis 1 as confirmed. This finding is particularly interesting because there are consistent data in the literature showing decreases in sexual function with aging [3], [4], [12], [13], [14]. Married couples experience the first impact on sexual activity between the fifth and sixth decade of life [6]. ILSE started at age 63, when participants had already adapted to the first change. We suppose that psychological adaptation processes according to the theory of Rothermund and Brandstädter about coping with deficits and losses in later life may play an important role for achieving stability [23]. The model of selective optimization with compensation (SOC) provides a framework to understand the specific mechanisms in the processes of coping [24]. We assume that at baseline of ILSE individual coping strategies – as a reaction to sexual changes experienced - had already started, thus resulting in a good level of satisfaction. The participants’ conditions for this adaptation process seemed favourable. They were in the so-called ‘third age’, characterised not only by good health and sufficient social, cognitive and physical activities, but also by high levels of cortical plasticity [25]. Further, a long period of cohabitation influences the way of coping with age-related physical and functional changes. Both previous relationships and partners' experiences during their lives determine perception and the reaction to it. It seems that particularly couples looking back on long-term relationships - like those of the ILSE-sample - could cope better with changes of sexual activity with age. A large number of years living together reflect both the maturity of the relationships and good choice of their partner. This could lead to mutual acceptance and feelings of worth within the relationship, something that could well alleviate negative experiences caused by physical and functional changes. Spouses are potential sources of emotional support especially when it comes to changes in sexuality. Familiarity and profound closeness built up over many years enables them to react favourably to changes and actively regain a fulfilling sexuality. In that sense the gap between satisfaction and activity is smaller among couples than among individuals without partners.

At measurement points 63 and 67 years satisfaction with sexuality was less in men than in women. However, at measurement point 74 years there were no longer gender-related differences. Hypothesis 2 can only be partially verified. Similar results with regard to satisfaction in women were described in a cross-sectional study of couples by Heiman et al [17]. We could speculate that possible processes of coping are probably moderated by gender and may explain the differences found here. The literature states that changes in female sexual functioning start earlier in life than those of men [26], [27], [28], [29], [30]. Furthermore, findings show that changes in sexual functioning are perceived as stronger and more stressful by men than women [3], [31], [32]. We can therefore assume that the emotional adaptation to the perception of changes in sexuality needs a longer period in men than in women.

Regarding hypothesis 3, we demonstrated that for older individuals affection is more important than sexual activity. 61% of 74 years old men stated that sexual activity is an important factor in their relationship, whilst only 27% of women said so. This corresponds to the findings of Waite et al. [3], but in addition to available results we found that affection is regarded as more important than sexual activity: 91% of men and 81% of women stated that affection was an important or very important part of their lives. Though no data were collected about a ’second language of sexuality‘ [33], which refers to elderly people experiencing a stronger emotional sexuality than younger persons and to the growing importance of various kinds of affection become more important in later life [34], this theory could help explain our findings. Affection in old age can be seen as an expression of strong intimacy and profound closeness. During their lives spouses have acquired the capacity to identify specific needs of their partner and react accordingly. Furthermore the importance of affection could be a reflection of the finiteness of life, particularly in the context of the relationship. We showed that both sexual activity and affection were more important for men than for women in long-term relationships. This effect of gender is well described [2], [35], [36], [37], [38]. The percentage of participants stating that sexual activity is important goes down with age both in men and in women. For men, however, it has been described that the importance plateaus after an initial decline, whilst the decline for women is constant [3]. Our findings demonstrate that the gender effect regarding the importance of sexual activity is not solely dependent on the higher risk of widowhood in women based on higher male mortality rates in contrast to female mortality rates. Also attitudes to and beliefs about sexuality in old age differ between men and women in long-term relationships [3].

Following the fourth hypothesis, we examined how satisfied participants were with their relationships. We found high levels of satisfaction in older age. Longitudinal studies show that compared to middle age adults, satisfaction with relationships is more pronounced in old age [39], [40], [41]. This can be explained by the ’socioemotional selectivity theory’: Older couples demonstrate more positive effective communication than middle-aged couples. In other words, over the years they have developed an ability to control the emergence of negative affects [42]. The regression analyses of predictors for satisfaction in relationships showed that neither education, physical health, nor the duration of the relationship could predict satisfaction. These results are consistent with the previous findings [17], [43]. Hypothesis four, predicting an influence of sexual activity and affection, could only be verified in part: only the importance of affection predicted satisfaction with the relationship. No support was found for the importance of sexual activity as predictor. Heiman et al. [17] demonstrate that men aged 40 to 70 years react to gestures of intimacy with high satisfaction in their relationship whilst in women no such correlation was found. However, we demonstrated that for women in the group 74 years, satisfaction with their relationship can be predicted by the role of day-to-day intimacy. We can therefore conclude that satisfaction with the relationship among older people depends on their openness and willingness to exchange intimacies. Elderly people want to satisfy their growing needs for mutual physical closeness by pleasant intimate daily rituals like the daily good morning/goodnight kiss, holding hands etc, all of which relatively independent of ‘with’, ‘instead‘ or ‘despite’ sexual activity. Individuals in stable and satisfactory relationships can use this ‘second language‘ with more nuances than the unsatisfied elderly.

## Limitations

Limitations are given by the specific protocol of exploration at MP 73 years. The sequence of the topics ‘affection’ and ‘sexuality’ and the different instructions to both may have affected the ratings, which slightly reduces comparability of both. Other limitations of our study are closely linked to the quantity and structure of the analysed sample. To study the course of satisfaction during the observation period of 12 years only subjects with complete data sets could be selected. Therefore the number of participants we could include in the study declined to194, which equates to 54% of all participants at the third measurement point. Death, separation or serious illness of a spouse were the most important reasons for non-responding. We therefore had to resort to a sub-sample of subjects almost exclusively living in long-term relationships. Our findings apply less to people living alone and mainly represent the elderly living in a relationship.

## Supporting Information

Table S1 **Data sheet containing all variables analysed.**
(PDF)
