# Supplementary material for: Sexuality and Affection among Elderly German Men and Women in Long-Term Relationships: Results of a Prospective Population-Based Study
Source: PLoS One. 2014 Nov 4;9(11):e111404. doi: 10.1371/journal.pone.0111404 (PMC4219747; doi:10.1371/journal.pone.0111404)
Supplement: Table S1 — Data sheet containing all variables analysed. (PDF) [file pone.0111404.s001.pdf]

sex	satisfaction_sexuality_63_years	satisfaction_sexuality_67_years	importance_affection_73_years	importance_sexuality_73_years	satisfaction_sexuality_73_years	education	age_second_followup	number_of_marriages	duration_relationship	physical_health_74_years	occupation	age_difference_spouses	age_first_follow_up	satisfaction_relationship_74_years	retirement_63_years	retirement_67_years	physical_health_63_years	physical_health_67_years	age_baseline
1	5	4	5	4	4	12	75	1	53	1	3	-39	67	5	2	1	1	3	62
1	2	4	5	4	4	11	75	1	53	4	6	48	67	5	1	1	4	4	63
1	3	4	4	4	4	16	75	1	52	2	6	-48	67	4	1	1	3	3	63
1	5	4	5	2	3	10	75	1	50	3	4	-84	67	5	1	1	3	5	63
1	2	4	5	4	5	12	73	1	43	2	7	-60	65	5	1	1	3	3	61
1	3	2	4	2	4	14	74	1	49	2	1	-7	66	4	2	1	2	3	62
1	1	3	2	3	2	13	75	1	47	2	5	-51	67	2	2	1	1	3	63
1	2	4	4	4	2	18	74	1	48	2	4	-52	66	2	2	1	2	2	62
1	1	4	4	4	4	12	74	1	50	4	6	39	66	5	1	1	2	4	62
1	4	4	5	3	4	13	74	1	40	1	1	-72	66	5	2	1	1	2	62
1	4	4	5	4	3	13	75	1	53	3	6	-1	67	5	1	1	3	4	63
1	3	4	4	4	4	14	75	1	52	4	5	6	67	5	1	1	3	4	63
1	3	5	5	4	5	14	74	1	48	2	4	-10	66	4	2	1	2	2	62
1	4	4	5	3	4	15	75	2	23	2	1	-106	68	5	1	1	1	2	63
1	4	4	5	2	4	12	74	1	51	2	1	-23	66	5	2	1	1	2	62
1	3	3	4	3	4	10	75	1	48	2	7	49	67	4	1	1	2	3	62
1	1	1	5	4	4	12	74	1	50	2	2	-72	67	4	1	1	3	3	62
1	5	5	5	3	5	13	75	1	47	2	2	-61	68	5	1	1	1	2	63
1	4	3	3	2	4	18	74	1	42	2	1	-24	67	4	2	1	2	3	63
1	5	3	4	4	3	13	73	1	54	3	5	-40	66	5	2	1	2	2	61
1	2	3	4	3	4	12	74	2	53	5	6	30	67	5	1	1	3	3	62
1	3	2	4	4	2	17	75	1	43	3	1	41	68	2	1	1	4	4	63
1	4	5	5	4	4	18	73	1	40	3	1	-84	66	5	1	1	2	2	62
1	2	4	5	4	2	16	74	2	24	3	1	-116	67	5	1	1	2	3	63
1	3	3	4	2	4	13	74	1	46	3	1	-93	67	5	1	1	2	3	63
1	2	2	5	4	1	16	75	2	37	4	1	-28	68	4	2	1	3	4	64
1	5	4	2	2	4	18	73	1	45	1	1	-76	66	5	2	1	1	2	62
1	3	2	4	3	4	12	75	1	48	3	6	-61	68	4	1	1	3	3	64

1	2	3	4	3	2	18	74	1	42	3	1	-61	67	5	1	1	2	2	63
1	3	2	4	4	4	12	76	1	51	2	6	-50	68	4	1	1	2	3	64
1	5	4	5	5	5	13	72	1	45	2	1	-47	65	5	2	1	2	2	61
1	4	4	4	2	4	18	74	1	44	4	1	-33	67	5	1	1	3	2	63
1	4	3	5	4	3	13	74	1	48	3	1	-24	67	5	1	1	2	2	63
1	3	3	4	4	2	14	73	1	53	2	1	-18	66	4	1	1	2	2	62
1	3	3	5	4	4	13	74	1	52	2	5	-21	67	5	1	1	2	2	63
1	4	3	5	5	5	12	75	1	52	1	2	-30	68	5	1	1	3	2	64
1	3	4	3	1	2	9	74	1	53	4	6	49	67	4	2	1	3	3	63
1	4	3	4	2	4	13	74	1	52	4	5	-2	67	4	1	1	3	4	63
1	2	4	3	3	3	18	73	1	45	3	1	-52	66	4	2	1	2	3	62
1	3	4	4	4	3	18	73	1	46	3	1	-22	66	5	1	1	2	2	62
1	5	5	5	3	4	13	73	1	45	2	4	-48	66	5	2	1	2	2	62
1	2	1	5	4	2	12	74	1	39	3	2	89	66	5	2	1	3	2	62
1	4	4	5	4	3	12	74	1	49	3	6	63	66	5	1	1	2	2	62
1	3	3	4	4	4	13	74	1	52	2	5	-5	67	5	1	1	2	2	63
1	2	1	4	4	1	12	75	1	54	3	6	2	68	1	1	1	3	3	64
1	3	2	4	4	3	12	73	1	42	2	6	-56	66	4	2	1	2	2	62
1	3	2	4	2	2	12	75	1	51	3	3	-133	68	4	1	1	2	3	64
1	2	3	4	4	5	11	74	1	48	3	1	13	67	5	1	1	3	3	63
1	4	4	4	2	4	12	74	3	21	3	2	-3	67	4	1	1	2	3	63
1	4	4	5	4	4	11	75	1	50	2	1	-58	68	5	1	1	1	2	64
1	5	4	5	5	4	12	75	1	51	3	1	8	68	5	1	1	4	3	64
1	4	5	4	2	4	18	74	1	42	3	1	-122	66	5	1	1	2	2	62
1	2	4	5	4	5	12	75	1	53	3	7	1	67	5	1	1	2	3	63
1	4	3	5	5	4	15	73	1	34	3	1	-162	66	5	2	1	2	2	62
2	3	4	4	3	4	12	75	1	28	2	5	37	69	5	1	1	2	2	63
2	4	4	4	2	4	12	74	1	52	3	1	24	66	4	1	1	2	3	63
2	1	1	4	4	1	13	73	1	50	3	7	56	65	4	1	1	4	3	61
2	4	5	5	4	5	14	74	1	55	3	1	-40	66	5	1	1	3	4	63

2	4	4	4	2	4	15	74	1	54	2	7	62	66	5	1	1	2	3	62
2	2	3	4	2	4	9	73	1	50	3	7	36	65	4	2	1	2	2	62
2	3	3	4	2	4	9	73	1	51	2	7	12	66	5	1	1	2	3	62
2	4	5	4	3	4	12	74	1	48	2	1	-12	67	5	1	1	1	2	63
2	3	5	2	1	4	18	73	1	47	2	1	22	66	3	1	1	3	3	62
2	3	4	4	2	4	12	75	1	51	3	7	7	68	4	1	1	3	2	64
2	4	4	5	4	4	18	75	1	47	2	1	-36	68	5	1	1	2	2	64
2	3	4	3	2	2	9	75	1	50	3	7	72	68	4	1	1	3	3	64
2	3	5	5	4	4	18	74	1	30	2	1	12	67	5	1	1	2	2	63
2	3	4	2	2	4	10	73	1	54	2	3	60	66	4	1	1	1	2	62
2	4	4	3	2	4	18	75	1	39	2	1	-132	68	5	1	1	2	2	64
2	4	4	5	2	4	12	74	1	54	2	7	96	67	5	1	1	3	3	63
2	3	4	4	3	3	9	73	1	31	3	7	-48	66	5	1	1	2	2	62
2	3	1	4	1	4	10	75	1	49	2	2	-11	68	4	1	1	2	3	64
2	4	4	5	3	4	13	75	1	53	2	7	168	68	5	1	1	2	3	64
2	4	4	3	3	3	12	75	1	55	4	2	36	68	4	1	1	2	4	64
2	4	4	4	2	4	9	74	1	54	3	4	48	67	4	2	1	1	2	63
2	4	4	4	4	4	9	73	1	51	2	3	24	66	5	1	1	1	2	62
2	3	1	2	2	3	9	74	1	48	2	3	-37	67	4	1	1	2	2	63
2	3	3	4	2	5	18	73	1	47	3	7	-7	66	4	1	1	2	2	62
2	1	3	4	2	3	9	74	2	24	3	7	22	67	4	1	1	2	2	63
2	4	4	5	2	4	10	74	1	53	2	3	3	66	5	2	1	2	2	62
1	2	5	4	4	4	14	75	1	52	1	4	-26	68	5	2	1	1	2	63
1	5	4	4	4	4	17	73	1	48	2	1	-84	65	4	1	1	2	2	61
1	3	4	4	4	4	13	74	1	53	2	3	1	66	4	1	1	3	2	62
1	3	4	4	3	4	14	73	1	51	2	2	36	65	3	1	1	2	2	61
1	3	4	4	4	4	14	75	1	42	2	1	-55	68	5	1	1	3	3	63
1	4	4	5	3	5	9	74	1	50	2	3	-76	65	5	1	1	3	2	61
1	4	3	4	2	4	18	75	1	55	3	2	2	67	4	2	1	2	2	63
1	4	4	4	2	4	18	75	1	53	3	1	60	67	4	1	1	3	2	63

1	4	4	4	4	4	18	75	1	47	3	1	-3	67	5	1	1	2	2	63
1	4	3	3	2	2	18	73	1	46	2	2	-39	65	5	1	1	3	2	61
1	4	3	5	4	5	18	73	1	52	2	1	13	65	5	1	1	3	2	61
1	4	4	4	3	3	12	74	2	46	2	6	-96	66	4	1	1	3	2	62
1	4	4	5	2	4	18	74	1	50	2	1	-36	66	5	1	1	3	3	62
1	4	4	4	4	4	14	73	3	25	2	1	-148	65	5	1	1	3	2	62
1	4	3	5	5	4	17	75	2	33	2	2	-192	67	5	1	1	3	3	64
1	4	4	5	4	5	12	75	1	55	3	6	24	67	5	1	1	2	2	64
1	5	5	4	4	2	12	73	1	52	2	1	13	65	5	1	1	2	2	62
1	4	4	4	3	4	13	74	1	54	4	1	2	65	4	1	1	5	3	62
1	4	4	5	2	4	12	73	2	34	2	4	-53	66	5	1	1	2	2	62
1	4	4	5	4	4	13	75	1	48	2	5	-84	68	5	1	1	2	2	64
1	3	2	5	2	4	14	73	1	50	2	1	24	66	4	1	1	2	3	62
1	1	1	4	4	2	18	75	1	49	2	2	-84	67	4	1	1	2	2	63
1	4	4	3	2	2	12	73	1	45	2	6	-11	66	4	1	1	2	2	62
1	3	1	5	5	2	13	75	2	26	3	3	-56	68	2	1	1	2	2	64
1	4	4	4	4	4	9	75	1	37	1	7	-200	68	4	1	1	2	2	64
1	5	4	5	5	4	12	75	2	47	3	6	-84	68	4	1	1	3	2	64
1	4	2	4	3	3	13	76	1	50	2	5	-72	68	4	1	1	2	3	64
1	4	4	3	3	4	18	75	1	49	3	2	-47	67	4	1	1	3	3	63
1	4	4	4	3	4	11	75	1	49	2	6	-65	68	5	1	1	2	2	64
1	4	2	4	2	4	18	74	1	49	2	1	-43	67	5	1	1	3	3	63
1	3	4	4	2	3	12	75	1	53	1	6	-17	68	5	1	1	3	2	64
1	4	5	4	4	1	18	74	1	49	2	1	-58	65	4	2	1	2	2	62
1	5	5	3	3	4	12	73	1	50	3	6	-28	66	5	1	1	3	2	62
1	2	4	5	5	3	14	75	1	52	2	1	-68	68	3	1	1	2	2	64
1	1	3	5	4	4	13	75	1	49	3	1	1	68	5	1	1	4	4	64
1	2	3	4	4	4	12	74	1	55	3	6	12	67	4	2	1	3	3	63
1	2	1	5	4	2	12	76	2	21	2	6	-93	68	5	1	1	2	3	64
1	2	4	5	5	5	12	75	1	54	2	5	-29	68	5	1	1	2	2	64

1	4	5	4	4	4	15	75	1	53	2	1	-19	68	5	1	1	2	2	64
1	1	3	5	4	2	16	75	1	48	2	1	-83	67	4	1	1	2	2	63
1	4	4	4	3	3	12	75	1	47	2	6	-38	68	4	1	1	1	2	64
1	4	4	3	2	4	13	73	1	45	2	1	-78	66	4	2	1	2	2	62
1	4	3	4	4	4	18	75	1	48	2	2	-81	67	4	2	1	1	2	63
1	4	4	5	4	4	12	73	1	49	2	6	-20	66	5	1	1	2	2	62
1	2	2	5	4	4	18	74	1	45	2	1	50	67	4	1	1	3	2	63
1	5	1	5	5	4	14	75	2	29	3	4	-158	68	5	2	1	2	3	64
1	5	3	5	4	5	12	74	3	21	2	6	-59	66	5	1	1	1	2	62
1	4	4	4	4	4	13	73	1	52	2	4	-25	66	4	2	1	2	3	62
1	1	4	4	4	4	12	74	1	50	2	6	1	66	5	1	1	2	2	62
1	4	4	4	2	4	12	75	1	52	3	6	-10	67	4	1	1	3	3	63
1	1	1	5	5	5	13	73	1	50	2	2	-31	66	5	1	1	2	2	62
1	2	1	4	4	4	16	73	1	53	3	1	-32	66	4	1	1	1	2	62
1	4	4	4	4	3	12	74	1	48	3	6	-16	66	4	1	1	2	3	62
1	3	4	5	4	4	14	73	1	51	2	1	-32	66	5	1	1	1	2	62
1	3	3	5	2	2	14	74	1	50	3	2	-25	66	4	2	1	2	2	62
1	4	4	5	5	2	14	75	1	48	3	1	-70	67	5	1	1	2	2	63
1	3	2	5	4	4	12	74	1	49	2	6	-26	67	5	2	1	2	2	63
1	4	2	4	4	4	13	74	1	51	3	5	-24	67	4	1	1	1	2	63
1	5	5	5	4	5	18	76	2	26	1	1	-102	68	5	1	1	1	2	64
1	4	4	4	2	4	18	74	1	46	2	1	-44	67	5	2	1	3	2	63
1	5	4	4	4	2	13	74	1	51	3	1	-2	66	4	1	1	2	2	63
1	2	3	4	3	3	18	75	1	31	2	1	-225	68	4	2	1	2	3	64
2	3	4	3	2	2	13	75	1	55	2	2	1	66	4	1	1	2	3	62
2	4	4	4	4	4	16	75	1	53	2	2	-13	66	5	1	1	3	3	62
2	4	3	5	4	2	14	75	1	55	3	5	86	67	4	1	1	3	3	64
2	4	4	5	4	4	18	74	1	52	2	2	86	66	5	1	1	2	3	63
2	2	3	4	4	4	13	74	2	38	2	3	120	65	4	1	1	2	3	62
2	4	4	5	2	2	13	73	1	47	2	3	-12	65	5	1	1	2	2	62

2	4	5	4	3	4	12	73	1	53	3	2	48	65	5	1	1	4	3	62
2	4	4	4	3	3	18	73	2	22	2	1	48	65	5	1	1	3	2	62
2	4	5	5	4	2	13	73	1	49	2	2	-27	66	4	1	1	2	2	62
2	3	4	2	1	3	12	73	1	55	2	6	36	66	2	1	1	2	4	62
2	4	4	4	3	4	12	75	1	53	3	2	-24	68	4	1	1	3	2	64
2	5	3	1	1	4	17	75	1	49	3	2	1	68	4	1	1	3	2	64
2	3	5	5	2	4	13	74	1	50	2	3	24	67	4	1	1	2	3	63
2	5	5	4	3	5	12	73	1	48	3	7	2	66	4	1	1	3	3	62
2	4	4	5	3	4	9	73	1	50	3	7	20	66	5	1	1	4	4	62
2	4	3	4	2	4	14	73	1	44	3	2	14	66	4	1	1	4	3	62
2	5	5	4	1	5	12	76	2	26	3	6	-29	68	5	1	1	2	2	64
2	5	4	4	2	4	12	74	1	51	3	3	15	66	4	1	1	4	3	62
2	5	5	5	4	4	9	74	1	50	2	3	48	67	5	1	1	4	4	63
2	4	4	4	3	4	12	76	1	56	3	6	-11	68	4	1	1	2	2	64
2	3	3	3	2	3	12	75	2	52	4	6	78	68	4	1	1	4	4	64
2	5	5	4	4	4	12	74	1	51	2	4	12	67	4	1	1	2	2	63
2	4	2	4	4	5	9	75	1	47	3	7	-76	68	5	1	1	2	2	64
2	5	4	5	1	4	12	74	1	52	2	2	108	65	5	1	1	2	2	62
2	4	3	5	4	4	18	74	1	52	2	1	-4	66	4	1	1	2	3	62
2	4	3	5	4	4	14	75	1	50	2	4	12	68	4	1	1	2	3	64
2	4	3	4	2	4	12	75	1	51	4	2	24	67	4	1	1	3	3	63
1	4	1	5	4	3	18	74	1	52	3	1	-1	67	5	1	1	2	3	63
1	1	4	5	4	3	18	73	1	51	3	2	-1	67	4	1	1	2	4	62
1	2	2	4	4	4	12	74	1	50	3	6	-39	68	4	1	1	2	2	64
1	4	4	5	3	1	17	72	1	48	3	3	-60	66	5	1	1	2	2	62
1	2	3	4	3	3	14	74	1	49	3	1	-27	68	4	1	1	3	2	64
1	3	3	4	4	3	12	73	2	24	3	1	-128	66	5	1	1	3	4	63
1	2	2	3	2	3	14	73	1	45	3	1	-36	67	4	2	1	2	3	63
1	2	3	4	4	3	15	73	1	50	3	1	108	67	4	1	1	2	2	63
1	3	3	4	3	3	14	74	2	36	3	1	1	68	5	1	1	3	3	64

1	2	1	4	3	1	18	73	2	49	4	1	-24	66	4	1	1	3	4	63
1	4	3	5	5	4	18	73	1	48	2	2	-10	66	5	1	1	1	1	63
1	1	3	4	4	3	18	72	1	47	2	1	-22	66	5	2	1	2	1	62
1	4	3	5	5	3	12	74	1	52	3	6	-20	67	5	1	1	2	2	64
1	4	5	4	4	2	12	72	1	50	3	3	-41	65	4	1	1	4	3	62
1	2	3	5	2	2	18	74	1	46	3	1	-78	68	5	1	1	3	2	64
1	3	2	3	3	3	14	74	1	52	2	1	72	68	4	1	1	2	1	64
2	5	3	4	3	3	12	74	1	49	2	6	1	67	4	1	1	4	2	63
2	5	3	4	4	4	13	73	1	55	3	3	1	67	4	1	1	4	3	63
2	3	4	3	2	3	13	74	1	52	2	1	24	67	4	1	1	2	3	64
2	3	3	4	2	4	18	74	2	51	2	1	156	67	4	1	1	2	2	64
2	4	4	5	2	4	18	74	1	50	2	1	-12	68	5	1	1	1	2	64
2	4	3	4	4	1	18	73	2	47	1	2	60	67	4	1	1	1	1	63
2	3	3	3	3	3	12	74	2	36	3	2	-54	67	4	1	1	3	3	64
2	4	3	4	2	3	18	73	1	49	4	1	12	66	4	1	1	2	3	63
2	4	1	4	2	1	17	72	1	51	3	2	60	65	4	1	1	4	2	62

variable label	value label
sex	1=“male“, 2=“female“
satisfaction_sexuality_63_years	1=”very poor”, 2=”poor”, 3=”satisfactory”, 4=”good”, 5=”excellent”
satisfaction_sexuality_67_years	1=”very poor”, 2=”poor”, 3=”satisfactory”, 4=”good”, 5=”excellent”
satisfaction_sexuality_73_years	1=”very poor”, 2=”poor”, 3=”satisfactory”, 4=”good”, 5=”excellent”
satisfaction_relationship_74_years	1=”very poor”, 2=”poor”, 3=”satisfactory”, 4=”good”, 5=”excellent”
importance_affection_73_years	1= “not at all important”, 2=”slightly important”, 3=”fairly important”, 4=”quite important”; 5=”very important”
importance_sexuality_73_years	1= “not at all important”, 2=”slightly important”, 3=”fairly important”, 4=”quite important”; 5=”very important”
physical_health_63_years	1=”very good”, 2=”good”, 3=”satisfactory”, 4=”sufficient”, 5=”poor”, 6=”very poor”
physical_health_67_years	1=”very good”, 2=”good”, 3=”satisfactory”, 4=”sufficient”, 5=”poor”, 6=”very poor”
physical_health_74_years	1=”very good”, 2=”good”, 3=”satisfactory”, 4=”sufficient”, 5=”poor”, 6=”very poor”
occupation	1="higher-grade professionals", 2="lower-grade professionals", 3="routine non-manual employees", 4="self-employed; artists", 5="employed technicians; supervisors", 6="skilled manual workers", 7="semi-skilled/unskilled manual workers"
retirement_63_years	1="yes", 2=“no“
retirement_67_years	1="yes", 2=“no“
